# Supplementary material for: Advanced paramedics’ restraint decision-making when managing acute behavioural disturbance (ABD) in the UK pre-hospital ambulance setting: A qualitative investigation
Source: PLoS One. 2024 May 16;19(5):e0302524. doi: 10.1371/journal.pone.0302524 (PMC11098407; doi:10.1371/journal.pone.0302524)
Supplement: Attachment — Submitted filename: Response to reviewers.docx [file pone.0302524.s001.docx]

8^th^ October 2023

Reference: PONE-D-23-16883

'This is on me.' A qualitative exploration of restraint decisions made by advanced paramedics in the UK when managing acute behavioural disturbance (ABD).
PLOS ONE

Dear Dr. Banerjee,

Thank you for your, and the reviewers’ feedback and the invitation to submit a revised version of our manuscript.

We have revised the paper and are pleased to re-submit this for your consideration. We outline our response to your, and the reviewers, points below.

**Editor’s Comments**

**Please ensure that your manuscript meets PLOS ONE's style requirements, including those for file naming.**

We have named the 3 updated files in accordance with PLOS ONE’s guidelines and the instructions in the revision letter. We have also checked through the manuscript to ensure it meets PLOS ONE requirements.

**We noted in your submission details that a portion of your manuscript may have been presented or published elsewhere. Please clarify whether this conference proceeding or publication was peer-reviewed and formally published. If this work was previously peer-reviewed and published, in the cover letter please provide the reason that this work does not constitute dual publication and should be included in the current manuscript.**

The draft abstract and poster to which we refer have not been and will not be subject to peer review. They have been submitted to the annual 999 Emergency Medical Services research conference, and the poster was displayed during that conference. The abstract has been omitted from publication of the proceedings.

**Reviewer’s Comments**

**1. Is the manuscript technically sound, and do the data support the conclusions?

The manuscript must describe a technically sound piece of scientific research with data that supports the conclusions. Experiments must have been conducted rigorously, with appropriate controls, replication, and sample sizes. The conclusions must be drawn appropriately based on the data presented. Reviewer #1: Yes Reviewer #2: Partly**

We have responded to individual reviewer comments below.

**2. Has the statistical analysis been performed appropriately and rigorously? Reviewer #1: N/A. Reviewer #2: No**

This is qualitative research and there is no statistical analysis. We trust that no further comment on statistical analysis is required.

**3. Have the authors made all data underlying the findings in their manuscript fully available? The PLOS Data policy requires authors to make all data underlying the findings described in their manuscript fully available without restriction, with rare exception (please refer to the Data Availability Statement in the manuscript PDF file). The data should be provided as part of the manuscript or its supporting information, or deposited to a public repository. For example, in addition to summary statistics, the data points behind means, medians and variance measures should be available. If there are restrictions on publicly sharing data—e.g. participant privacy or use of data from a third party—those must be specified.** **Reviewer #1: No Reviewer #2: No**

Anonymised transcripts are available via the University of Bath Research Data Archive. The data relates to a small professional group and a controversial subject. The data is available to researchers on application, rather than on an open access basis. This is to protect the privacy of participants and this requirement forms part of the ethical approval for this study. The reference and DOI for the data will is as follows: Lindridge, J., Edwards, T., Blackwood, L., in press. Dataset for "Advanced paramedics' restraint decision-making when managing acute behavioural disturbance (ABD): A UK based qualitative investigation.". Bath: University of Bath Research Data Archive. <https://doi.org/10.15125/BATH-01326>. The DOI will be activated on publication of the manuscript.

**4. Is the manuscript presented in an intelligible fashion and written in standard English?

PLOS ONE does not copyedit accepted manuscripts, so the language in submitted articles must be clear, correct, and unambiguous. Any typographical or grammatical errors should be corrected at revision, so please note any specific errors here. Reviewer #1: Yes Reviewer #2: No**

We have responded to individual reviewer comments below.

**5. Review Comments to the Author

Reviewer #1: Thank you for meaningful and insightful work. May i provide some suggestions for your considerations.

The Title starting with "That is on me." It could be leading the readers to seme aspects of the APs' perceptions or psychological impact, etc. The statement could not reflect or conclude all the themes developed. Therefore, suggest removing it.**

We would like to thank Reviewer 1 for their engagement with our research and for their positive comments. We also appreciate the guidance they have provided. We agree that the title might be misleading and thank the reviewer for bringing this to our attention. We have now revised the title to: Advanced paramedics' restraint decision-making when managing acute behavioural disturbance (ABD): A UK based qualitative investigation.

**Introduction and background: overall, the general sensing for the background is that some inferences allude there are needs or gaps to be addressed for the topic of APs' decision-making in restraints for people with ABD. However, the big proportion of the literature review were discussing concerns of paramedics, not APs, and in management of other conditions than ABD. Hence, the relevancy of the review could not very convincing. Suggest relooking into adding more specific background of APs, your targeted population, in the specific group of ABD.**

We are grateful for this feedback. There is very little literature on APs and accordingly we have needed to draw on wider literature which we believe does have relevance. We agree this was not clear in the previous manuscript; a paragraph outlining the rationale for including literature relating to paramedics in the literature review has been included to make the relevance of this research included much clearer. We have also now specifically described the relevance of evidence cited to APs, we hope this addresses the reviewer’s concerns.

**Line 75, "Research has identified that paramedics have felt unprepared by their formal training", what about the APs? was it due to ineffective training?**

As noted above, there is little literature on AP’s experiences so we cannot comment on the extent to which this is also true for this group. We have amended the manuscript to make this clearer, and are grateful to the reviewer for bringing this to our attention.

**Line 82, other heathcare professionals also rely on tacit knowledge when making restraint decisions." Is it a common problem in all professionals handling restraint. so, what is unique need to look into the APs?**

Our literature review did not explicitly explore whether relying on tacit knowledge is common to all or most healthcare professionals. The relevance to looking specifically at APs is related to their extended scope of practice, which includes forms of chemical restraint. We have amended the manuscript to make this clearer, we are grateful for the feedback.

**Line 84, since there was paucity of research, how did you gather there are concerns/gaps to address in the APs' restraint process?**

We thank reviewer 1 for your highlighting this omission. We determined that there were concerns and gaps to address because this area has been highlighted as a matter of concern by coroners, regulators and the professional body for paramedics in the UK. We have included more information in the introduction to explain this, as well as highlighting a scoping review into patient safety in ambulance service which also identified the need for more research in this area.

**There might be a need to define "clinician", as it might mean different scope of role in some countries? are they doctors?**

We are grateful to reviewer 1 for highlighting this, and we agree that the definition of clinician is likely to vary internationally. We have added a definition so it is clear to readers how we are interpreting this term and the types of healthcare worker it relates to in the UK pre-hospital ambulance setting.

**Are paramedics and APs used interchangeably in the manuscripts? As your target population is APs, Line 84-125, concerns of paramedics were discussed. Do APs face similar issue?**

We agree a clearer distinction between paramedics and APs would be helpful and thank reviewer 1 for bringing this to our attention. The relevance of paramedic research on APs has been made clearer throughout this section, with explicit explanations on how literature is applicable to APs where appropriate. We hope this has improved the manuscript and addresses these concerns.

**Philosophy: the adoption of critical realist combines ontological realism and epistemological constructivism is a great application in this topic. To facilitate better understanding by a wider group of readers, it would be great to elaborate on how its applied.**

We thank reviewer 1 for the feedback. Further detail relating to critical realism has been added to this section to explain how we have applied this philosophy to the phenomenon under study, in that we take the position that the decisions made in this context are real and our participants make sense of that reality in a social constructed way. We hope this has enriched the manuscript.

**Sampling: seven out of ten volunteers were selected. why the three are excluded? will be any chance the insider researcher could be asking leading questions? how is it avoided?**

In relation to sampling, the 3 potential participants were not specifically excluded but rather did not follow up on their expression of interest. Accordingly, we have removed reference to these three. In relation to questioning, participants were invited to tell stories and to give accounts of events that were meaningful to them. This was done to ensure that the participant could raise what they thought was important. The interviewer was aware of her insider status and the risks involved, she addressed this through open ended questions and member checking. We have provided additional information on how the interview was approached which we hope will address the reviewer’s concerns.

**Data collection and processing: how the informal member checking is sone? author TE did not have access to information that could identify the participants. what is the implication of this?**

In relation to member checking, this was achieved by the interviewer restating points in the interview and inviting the participants to confirm accuracy of understanding. A note to explain this has been added. In relation to TE's access to participant personal data, only one supervisor (the academic supervisor) required access to participant personal data. This has been clarified in the text. Access to participant personal data was necessary for TE’s role as practice-based supervisor, and there is no implication of this in relation to data analysis.

**Analysis: Line 393 "psychological safety" might not be very suitable to use here, as it could be caused by a range of factors. suggest rephrasing it, e.g. impact of employer support, or organizational support.**

We thank reviewer 1 for the feedback. We have amended the manuscript accordingly, replacing ‘psychological safety’ with ‘employer assistance and support.

**Reviewer #2: In the current study, seven semi-structure in-depth interviews have been reported and authors have used Braun and Clarke’s suggestion as their argument for having small sample size. However, it has not been justified properly. Therefore, it is suggested to collect more data or present sufficient rationale for choosing such small sample size.**

We are grateful to reviewer 2 for this feedback and agree that this point would benefit from additional justification. We have amended the sampling and recruitment section to include more information on our rationale for determining the final sample size. We determined that the participants had a high level of experience of the phenomenon under study, and the interview dialogue was of high quality. Consequently, we agreed that seven interviews generated sufficient rich and complex data to generate meaningful theme. We trust this addresses reviewer 2’s concerns.

**Please also provide demographic information of your sample.**

This is provided in the 'participants' section, this has been augmented to note all of the participants are of adult working age.

**In the manuscript, many technical terms have been used but they have not been defined properly and restructuring some sentences. The manuscript requires language revisions to improve readability and meaning of the text.**

We thank reviewer 2 for this feedback. We have reviewed the manuscript and clarified technical terms throughout, as well as reviewing language and syntax throughout, making revisions throughout.

**The rationale of the study is mentioned but for better structure and organization, please provide rationale of the study as a separate subsection.**

We thank reviewer 2 for their comments and agree the structure and organisation of the manuscript could be improved. We have added a ‘research rationale’ subsection accordingly.

**The term ‘Philosophy’ is an ambiguous term. Please replace it with appropriate sub-heading.**

We thank reviewer 2 for their comments and have amended this sub-heading to 'research philosophy'.

**It will be better if you could give the demographic info (age and sex) of the respondents along with their excerpts.**

The demographics of participants are provided in a separate section. This section explains that of the seven participants, only one was female and highlights that to protect anonymity, gender-neutral pseudonyms were used for all participants. At the time of the study, the majority of APs in the population were male, and the sample represented approximately 18% of the total population, therefore there is a risk of inadvertently identifying participants without careful anonymisation. The research team remain of the view that including the gender of participants along with their excerpts represents a risk to participant anonymity.

**The result section seems confusing. It should not be named ‘analysis’ since this section talks about the findings. Moreover, it should be restructured for more clarity. It seems that themes are given within the context of participants. It should be made clear from the beginning.**

We thank reviewer 2 for their feedback. We have re-titled the analysis section 'findings'. In relation to the structure of this section, this is organised as a thematic analysis and therefore consists of a balance of data extracts from participants and analytic narrative. A explanation of this has been added at the beginning of this section which is intended to assist the reader navigate the analysis which follows, and make this clearer. We hope this addresses the reviewer’s concerns.

In conclusion, we hope you find our amendments to be satisfactory, and we look forward to hearing from you.

Kind regards

Ms Jaqualine Lindridge
