# Supplementary material for: Advanced paramedics’ restraint decision-making when managing acute behavioural disturbance (ABD) in the UK pre-hospital ambulance setting: A qualitative investigation
Source: PLoS One. 2024 May 16;19(5):e0302524. doi: 10.1371/journal.pone.0302524 (PMC11098407; doi:10.1371/journal.pone.0302524)
Supplement: Attachment — Submitted filename: Response to reviewers.vRev2.1.docx [file pone.0302524.s002.docx]

17^th^ February 2024

Reference: PONE-D-23-16883

'This is on me.' Advanced paramedics restraint decision-making when managing acute behavioural disturbance (ABD) in the UK pre-hospital ambulance setting: A qualitative investigation.
PLOS ONE

Dear Dr. Bastani,

Thank you for your, and the reviewers’ feedback and the invitation to submit a revised version of our manuscript.

We have revised the paper and are pleased to re-submit this for your consideration. We outline our response to your, and the reviewers, points below.

**Reviewer’s Comments**

**4. Have the authors made all data underlying the findings in their manuscript fully available? The PLOS Data policy requires authors to make all data underlying the findings described in their manuscript fully available without restriction, with rare exception (please refer to the Data Availability Statement in the manuscript PDF file). The data should be provided as part of the manuscript or its supporting information, or deposited to a public repository. For example, in addition to summary statistics, the data points behind means, medians and variance measures should be available. If there are restrictions on publicly sharing data—e.g. participant privacy or use of data from a third party—those must be specified.** **Reviewer #3: No**

Anonymised transcripts are available via the University of Bath Research Data Archive. The data relates to a small professional group and a controversial subject. The data is available to researchers on application, rather than on an open access basis. This is to protect the privacy of participants and this requirement forms part of the ethical approval for this study. The reference and DOI for the data will be as follows: Lindridge, J., Edwards, T., Blackwood, L., in press. Dataset for "Advanced paramedics' restraint decision-making when managing acute behavioural disturbance (ABD): A UK based qualitative investigation.". Bath: University of Bath Research Data Archive. <https://doi.org/10.15125/BATH-01326>. The DOI will be activated on publication of the manuscript.

**5. Is the manuscript presented in an intelligible fashion and written in standard English?

PLOS ONE does not copyedit accepted manuscripts, so the language in submitted articles must be clear, correct, and unambiguous. Any typographical or grammatical errors should be corrected at revision, so please note any specific errors here. Reviewer #3: No**

We have responded to individual reviewer comments below.

**6. Review Comments to the Author**

We would like to thank Reviewer 3 for their engagement with our research and for their time in preparing this helpful review.

**1. The context of this study targeted ABD incidents in the UK pre-hospital ambulance setting and it is hoped that reflecting this context in the title, will help the reader to clarify the topic of the study. Because in some countries and regions, the reference to paramedics may not be immediately associated with pre-hospital ambulance service.**

Thank you for alerting us to this, we agree and have amended the title, and short title, accordingly. The title now reads ‘Advanced paramedics’ restraint decision-making when managing acute behavioural disturbance (ABD) in the UK pre-hospital ambulance setting: A qualitative investigation.’

**2. line 18, the aim of the study in the abstract should also highlight that the study setting is ABD incidents in the UK pre-hospital ambulance setting.**

Thank you for highlighting this omission, this has been amended accordingly at line 22 with ‘The aim of this research is to explore the decisions made by APs when managing restraint in the context of ABD, in the UK pre-hospital ambulance setting.’

**3. Line 28, hoping to show that the themes are summarized from the interviews.**

We have clarified in the abstract that the themes arose from the interview data, now at line 27.

**4. A larger proportion of the literature review refers to paramedics, with less mention of Aps in this study, and the authors mentioned fewer Aps-related studies in the previous peer review, but an appropriate increase in the content of the literature review to include a description of Aps would be beneficial.**

Thank you for highlighting the need for further work on this section. We included a statement at line 130 to make it clearer that no studies specifically relating to APs were identified. We have also included an acknowledgement of several included studies which reported data from specialist or enhanced paramedics in addition to general paramedics (line 101). Whilst not working at an advanced level, specialist/ enhanced paramedics typically hold additional qualifications and work at a more enhanced scope of practice than general paramedics. We have also moved and refined the definition of paramedics and APs from earlier in the manuscript to line 69 in response to this feedback. We hope this addresses your concern and improves the readability of the manuscript.

**5. The content after the abstract and before the methods is very similar to the introduction. The authors may have written in points for the purpose of a better structure. But as far as the content seems confusing. It is recommended that the Introduction and background, Definitions, Literature review, and Research rationale be integrated into a new Introduction and background. When integrating, if the writer tends to write in points, then it is recommended that Definitions, Literature review, and Research rationale be categorized under the general heading of Introduction and background, which may be beneficial for readability.**

Thank you for highlighting this issue. We agree the structure could be improved. To address this, we have redrafted the content between the abstract and methods section (lines 46-144). We have removed the additional headings (definitions, literature review and research rationale) and the content is now presented in a single ‘Introduction and methods’ section. We have removed repetition, and integrated the content into a single section, which is slightly shorter as advised. We hope this improves the readability of the manuscript and satisfactorily addresses your feedback.

**6. In line 86, advanced paramedics shorthand APs have been provided in the text, but there are a large number of advanced paramedics in the text (e.g. lines 90, 100, 137).**

Thank you for highlighting this. We have reviewed the entire manuscript and made several corrections. The shorthand ‘AP’ is now used consistently throughout and is written in full only on its first use (line 58).

**7. Line 234, Paramedics (including APs), recommendation to delete Paramedics.**

Thank for highlighting this, we agree it is unhelpful to refer to both paramedics and APs here and have deleted ‘paramedics’ as recommended. Now at line 196.

**8. The sample size is indeed a concern, and it is worth noting that the authors have responded to this in the previous peer review, but there is still some concern about the sample size, and it is hoped that pointed out in the limitations.**

Thank you for highlighting this and acknowledging our response to previous peer review. We have now added a sentence in the limitations section at line 675: ‘This was a small study in nomothetic, statistical terms, but not in idiographic terms where the focus was understanding experiences and sense-making within a small community of professionals’. We have also provided a reference to a recent systematic analysis of justifications for sample size.

**9. The manuscript still has paramedics and APs used interchangeably, which seems confusing.**

We have reviewed the entire manuscript to identify occasions where paramedics and APs were used interchangeably and made corrections throughout, particularly in our analysis where we agree references to ‘paramedics’ when analysing the data contributed by APs was confusing. The term ‘paramedic’ is now used much less frequently and much more specifically throughout the manuscript. Thank you for drawing this matter to our attention.

In conclusion, we hope you find our amendments to be satisfactory, and we look forward to hearing from you.

Kind regards

Ms Jaqualine Lindridge
